# Identification of lipid metabolism-related genes in dapagliflozin treated rats with diabetic cardiomyopathy by bioinformatics

**DOI:** 10.3389/fendo.2025.1525831

**Published:** 2025-03-20

**Authors:** Xun Huang, Yunhong Wang, Rong Wan, Zhigang You, Lin Huang

**Affiliations:** ^1^ Department of Cardiovascular Medicine, The Second Affiliated Hospital of Nanchang University, Nanchang, Jiangxi, China; ^2^ Department of Cardiology, Ningdu County People’s Hospital, Ganzhou, Jiangxi, China; ^3^ Jiangxi Key Laboratory of Molecular Medicine, The Second Affiliated Hospital, Jiangxi Medical College, Nanchang University, Nanchang, Jiangxi, China

**Keywords:** diabetic cardiomyopathy, dapagliflozin, lipid metabolism, hub gene, bioinformatics analysis

## Abstract

**Background:**

Diabetic cardiomyopathy (DCM) is a heart disease caused by the metabolic disorders of glucose and lipids associated with diabetes, leading to heart failure and death in diabetic patients. Dapagliflozin (DAPA) serves as a treatment for managing blood glucose levels in individuals with type 2 diabetes mellitus (DM). However, the specific mechanisms by which DAPA treats DCM are not yet fully understood.

**Methods:**

Sprague-Dawley (SD) rats (n = 5/group) were randomly divided into control, model, and intervention groups. Lipid metabolism-related genes (LMRGs) were gotten from publicly available database. Differential expression analysis of model *vs.* control and intervention *vs.* model samples was performed to obtain differentially expressed genes (DEGs), and the result was recorded as DEGs-Model and DEGs-Intervention. The intersection of genes with opposing expression trends between DEGs-Model and DEGs-Intervention were considered as candidate genes. Subsequently, candidate genes and LMRGs were intersected to acquire hub genes, and the expression of hub genes was analyzed in each group of samples. Then, the mechanism of action of these hub genes were investigated through functional enrichment analysis, gene set enrichment analysis (GSEA), and predictive of m6A binding sites.

**Results:**

Ultimately, 68 candidate genes and 590 LMRGs were intersected to derive 2 hub genes (Acsbg1 and Etnppl). Acsbg1 was significantly increase in model group compared with control group. RT-qPCR results confirmed Acsbg1 was obviously higher expression in model group, while Etnppl was significantly lower expression in model group compare to control groups and intervention group. While the expression of Etnppl was significantly increase in intervention group compared with model group. Functional enrichment analyses indicated that Acsbg1 and Etnppl were associated with fatty acid metabolism. The findings of GSEA indicated that Acsbg1 and Etnppl might affect the occurrence and progression of DCM through lysosome. And the Acsbg1 and Etnppl were located at UCAGG in the RNA secondary structure.

**Conclusion:**

This study identified 2 hub genes (Acsbg1 and Etnppl) as potential new focal points for diagnosing and treating DCM.

## 1 Introduction

Diabetic cardiomyopathy (DCM) is a distinct form of heart disease that occurs in patients with diabetes mellitus (DM), independent of traditional cardiovascular risk factors like hypertension, coronary artery disease, and valvular diseases (1), the prevalence of heart failure among diabetic patients globally ranges from 19% to 26%, DCM is the leading cause of heart failure in diabetic patients (2). DCM primarily arises from chronic hyperglycemia and insulin resistance, leading to a maladaptive shift in cardiomyocyte energy metabolism from glucose oxidation to excessive reliance on fatty acid (FA) oxidation. This metabolic shift, coupled with lipotoxicity-induced cellular damage, inflammatory responses, activation of the advanced glycation end products receptor for advanced glycation end products pathway, myocardial fibrosis, and impaired calcium homeostasis, culminates in structural and functional myocardial alterations, ultimately causing DCM (3, 4). Due to the incomplete understanding of the complex molecular mechanisms underlying DCM, developing reliable therapeutic targets and effective pharmacological interventions remains challenging (5). Therefore, exploring hub genes in DCM is crucial for understanding its exact mechanisms and developing new strategies to reduce the risk of DCM patients.

Lipid metabolism dysregulation plays a fundamental role in the pathogenesis of DCM (6). Lipid metabolism disorders lead to an increase in lipid peroxidation products, which further exacerbate oxidative stress and inflammatory responses (7). The involvement of peroxisome proliferator-activated receptor (PPAR) in diabetes-related metabolic disturbances and lysosomal abnormalities can impair lipophagy, the autophagic process that utilizes lipids as substrates, leading to intracellular lipid accumulation (1, 8). This is believed to significantly contribute to the development of DCM. Consequently, a more in-depth investigation into how lipid metabolism disorders contribute to DCM could offer new theoretical insights and identify potential targets for effective treatment of this condition.

Sodium-glucose cotransporter-2 (SGLT2) inhibitors, a novel class of glucose-lowering medications, reduce plasma glucose PG by producing glycosuria. significantly reduce hospitalization and mortality rates in patients with heart failure (9). SGLT2 inhibitors not only help lower blood sugar but also assist patients with type 2 diabetes in losing weight, reducing blood pressure, and decreasing the risk of cardiovascular events. SGLT2 inhibitors may promote FA oxidation and reduce fat accumulation in tissues by activating AMP-activated protein kinase (AMPK) and increasing the expression of fibroblast growth factor 21 (FGF21) (10). Dapagliflozin (DAPA) is one of the earliest SGLT2 inhibitors used for glycemic control in patients with type 2 diabetes mellitus (11). In obesity-related cardiomyopathy (HFD), DAPA can significantly reduce body weight and improve lipid levels (12). Besides its lipid-lowering effects, DAPA provides direct protective effects against myocardial cell damage induced by saturated FA However, the specific biological mechanisms of DAPA treatment in DCM are currently unclear.

To investigate the hub genes involved in lipid metabolism during DAPA treatment for DCM, this study used DCM rats as a model. Bioinformatics analysis was employed to examine the biological pathways these hub genes participate in, and the molecular regulatory network was explored. The aim is to provide new reference points for clinical treatment research of DCM.

## 2 Materials and methods

### 2.1 Establishment of DAPA-intervened DCM model

The 15 male Sprague-Dawley (SD) rats (180 g-220 g, 6-8 weeks old) were obtained from the Beijing Huafukang Bio-technology Co. (Production License No.: SCXK (Beijing) 2019-0010; Use License No.: SYXK (Dian) K2020-0006). The Ethics Committee of the Second Affiliated Hospital of Nanchang University granted ethical approval (Approval No. NCULAE-20221031151). The rats were randomly assigned to one of three groups: the control group (n = 5), the DCM model group (n = 5), and the DCM + DAPA intervention group (n = 5). Rats in the control group were fed a normal diet, while rats in the model and intervention groups were fed a high-fat, high-sugar diet for 4 weeks. After this feeding period, insulin resistance was assessed using the peritoneal glucose tolerance test (IPGTT) and insulin tolerance test (IPITT). To induce DM in the model and intervention groups, rats were given a single intravenous injection of streptozotocin (STZ, 35 mg/kg). Control rats received an intraperitoneal injection of citrate buffer at the same time. Fasting blood glucose levels were measured from the tail vein 3 and 7 days after the STZ injection. Rats with fasting blood glucose levels ≥ 11.1 mmol/L were considered successfully induced DM. Following successful DM induction, rats in the DCM and DCM + DAPA groups continued to receive the high-fat, high-sugar diet, while the control group was maintained on a normal diet. After 8 weeks, echocardiography was performed, confirming the presence of left ventricular diastolic dysfunction in both the model and intervention groups, indicating successful induction of DCM. After DCM model was confirmed, rats in the DCM + DAPA group were administered dapagliflozin (DAPA) at a dose of 1 mg/kg/day in their drinking water, while rats in the DCM group received no treatment. Following 6 weeks of drug intervention, all rats were euthanized under anesthesia according to ethical guidelines, and myocardial tissue samples were collected from the left ventricle for further analysis.

### 2.2 Hematoxylin and eosin staining

HE staining is a fundamental technique in histology and pathology for visualizing tissue samples under a microscope. Tissue sections (5 µm thick) were prepared from paraffin-embedded specimens that had been fixed in 4% paraformaldehyde (PFA). After baking at 64°C for 1 h, the tissue sections were deparaffinized in xylene and then rehydrated through a graded ethanol series (100%, 95%, 80%, and 70%) to remove the xylene and gradually restore the tissue’s water content. After rehydration, the sections were stained with hematoxylin for 5 min, followed by a wash in running tap water to differentiate the stain. The sections were then stained with eosin for 10-15 s to highlight cytoplasmic components. Finally, the slides were dehydrated through a graded ethanol series (95% and 100%), cleared in xylene, and mounted with a coverslip using a mounting medium for microscopic examination. Finally, the stained tissue sections were examined under a light microscope for histological analysis.

### 2.3 Masson staining

Masson’s staining effectively highlights collagen fiber changes, enabling assessment of collagen proliferation and the progression of DCM. First, prepare Weigert’s iron hematoxylin solution by mixing Reagents A1 and A2 in a 1:1 ratio just before use. Apply the solution to tissue sections for 5-10 min, then rinse with distilled water. Differentiate with acid ethanol for 5-15 s, followed by a 30 s wash in distilled water. Next, applying Masson’s blueing solution for 3-5 min, then rinse with distilled water (30 s). After, Biebrich Scarlet-Acid Fuchsin was utilizing to stain for 5-10 min. During staining, prepare the weak acid solution by mixing distilled water and weak acid in a 2:1 ratio. Following this, apply the weak acid solution to the sections and wash for 30 s. Drain excess liquid and apply phosphomolybdic acid for 1-2 min, followed by a 30 s wash with the weak acid solution. Clear the sections in xylene twice, for 1-2 min each, then mount with neutral balsam. Finally, the proliferation of collagen fibers in the tissues was observed and captured in images.

### 2.4 Source of data

Lipid metabolism-related genes (LMRGs) were gotten from Molecular Signatures Database (MSigDB, https://www.gsea-msigdb.org/gsea/msigdb): Using “lipid metabolism” as the keyword, gene-ID was converted to rat gene, and then 590 LMRGs were obtained by removing duplicate genes (**Supplementary Table S1** **) (** 13).

### 2.5 Transcriptome sequencing and data preprocessing

Total RNA was isolated and purified from tissue samples using TRIzol (Invitrogen, CA, USA). Next, the quantity and integrity of the total RNA were assessed using a NanoDrop ND-1000 spectrophotometer (NanoDrop, Wilmington, DE, USA) and a Bioanalyzer 2100 system (Agilent, CA, USA), respectively. Samples with concentrations > 50 ng/µL, RIN values > 7.0, OD 260/280 > 1.8, and total RNA > 1 µg were considered suitable for downstream experiments. Then, Poly(A) RNA was purified from 1 µg of total RNA using Dynabeads Oligo (dT)25-61005 (Thermo Fisher, CA, USA) through two rounds of purification. Subsequently, the poly(A) RNA was fragmented into small pieces using the Magnesium RNA Fragmentation Module (NEB, cat. e6150, USA) at 94°C for 5-7 minutes. The cleaved RNA fragments were then reverse-transcribed to create cDNA using SuperScript™ II Reverse Transcriptase (Invitrogen, cat. 1896649, USA). What’s more, Polymerase Chain Reaction (PCR) amplification was performed under the following conditions: initial denaturation at 95°C for 3 minutes; 8 cycles of denaturation at 98°C for 15 seconds, annealing at 60°C for 15 seconds, and extension at 72°C for 30 seconds; followed by a final extension at 72°C for 5 minutes. The average insert size for the final cDNA library was 300 ± 50 bp. Sequencing was carried out on the Illumina NovaSeq 6000 platform using the paired-end 150 bp (PE150) sequencing mode.

Afterwards, data quality was assess using FastQC (v 0.11.9), and low-quality data were filtered using Trimmomatic (v 0.39). For mRNA sequencing reads alignment, clean data was mapped to the reference genome (Rat Rnor 6.0 genome) using the alignment tool hisat2 (v2.2.1) with default parameters. Finally, a gene expression matrix was generated for subsequent analysis.

### 2.6 Base mass value and content distribution of sequencing data

Phred scaled quality value (Q value) was used to assess the base error probability. The Q value was inversely proportional to the probability of error, and when the Q value reached 30, the probability of a base being in error was only 0.001, meaning that only about 1 out of every 1,000 bases was likely to be in error. In addition, the distribution of base types in the sequencing data was analyzed, particularly with regard to the proportion of guanine (G) as well as cytosine (C) in the DNA sequences. In general, a uniform distribution of GC content means that the data have not been affected by exogenous DNA contamination or other technical factors.

### 2.7 Comparison of transcriptome data with reference genome sequences

To understand more deeply the comparison of the transcriptome data with the reference genome sequence, the study utilized a designated reference genome for alignment analysis. Firstly, the sequences in the transcriptome data were aligned with the reference genome sequences to determine their positions in the genome. Then, the successfully aligned sequences were assembled and quantitatively analyzed using StringTie software (v 2.1.6) to probe gene expression and gene structure. The analysis focused on the distribution of the sequences in different regions of the genome (e.g. exons, introns, and intergenic regions) (14).

### 2.8 Gene expression analysis

Next, the fragments per kilobase transcript model per million mapped fragments (FPKM) was calculated to measure the expression level of transcript or gene by the StringTie software (v 2.1.6). The FPKM standardization method takes into account differences in sequencing depth and gene length to make gene expression comparable across samples. The number of sequences mapped to the genome and the length of transcripts in each sample were normalized to calculate FPKM values for each gene. Then these FPKM values were log10 transformed to better demonstrate the distribution of gene expression.

### 2.9 Identification of differentially expressed genes

In order to assess the variability in the expression of the transcriptome data of the 3 groups of samples, the transcriptome sequencing data was analyzed via principal component analysis (PCA). Differential expression analysis of model *vs.* control and intervention *vs.* model samples was performed to obtain DEGs-Model and DEGs-Intervention by the “DESeq2” R package (v 1.34.0) (15) (|log_2_Fold Change (FC)| > 0.5 as well as p.value < 0.05). Volcano maps as well as heat maps of the DEGs-Model and DEGs-Intervention were visualized via “ggplot2” R package (v 3.4.3) (16) and “ComplexHeatmap” R package (v 2.14.0) (17).

### 2.10 Identification and functional enrichment analysis of candidate genes

The intersection of up-regulated genes in DEGs-Model with the down-regulated genes in DEGs-Intervention and down-regulated genes in DEGs-Model with the up-regulated genes in DEGs-Intervention were recorded as candidate genes via “ggvenn” R package (v 0.1.9) (18). To elucidate the potential biological functions and related pathways, Gene ontology (GO) enrichment analysis was performed on candidate genes (p.value < 0.05). The GO enriched results were sorted by P.value from smallest to largest, and the top 10 significant pathways were shown via “GOplot” R package (v 1.0.2) (19). Kyoto Encyclopedia of Genes and Genomes (KEGG) was performed on candidate genes (p.value < 0.05). The KEGG enriched results were sorted by count from smallest to largest, and the top 10 significant pathways were shown by “clusterProfiler” R package (v 4.7.1.003) (17).

### 2.11 Identification and functional enrichment analysis of hub genes

Candidate genes and LMRGs were intersected to acquire hub genes, while the expression of hub genes was analysed in each group of samples via “ggvenn” R package (v 0.1.9). Then, GO enrichment analysis was performed on hub genes (p.value < 0.05). The GO enriched results were sorted by p.value from smallest to largest, and the top 10 significant pathways were shown by “GOplot” R package (v 1.0.2). And KEGG was performed on hub genes (p.value < 0.05). The KEGG enriched results were sorted by count from smallest to largest, and the pathways were shown by “clusterProfiler” R package (v 4.7.1.003). Gene-gene interaction (GGI) network was constructed by GeneMANIA (http://www.genemania.org/), and the network interactions of hub genes and the biological functions involved were explored at the protein level.

### 2.12 Gene set enrichment analysis

The background gene set (“c2.cp.kegg_medicus.v2023.2.Hs.symbols.gmt”) was downloaded from the MSigDB. The Spearman correlation coefficients between the expression levels of hub genes and other genes were ranked by “psych” R package (v 2.1.6) (20), and then the GSEA was performed through “clusterProfiler” R package (v 4.7.1.3) (21) (|normalized enrichment score (NES)| > 1 as well as adj. p < 0.05), and the pathways ranked in the top 5 in the |NES| were selected for presentation.

### 2.13 Predictive analysis of m6A binding sites

To investigate whether m6A affects the translational stability of hub genes, the m6A binding sites of hub genes were predicted. Sequence files of the hub genes were acquired from National Center for Biotechnology Information (NCBI, https://www.ncbi.nlm.nih.gov/gene/). The m6A modification sites of the hub genes and their locations in the RNA secondary structure were predicted by the sequence-based RNA adenosine modification site predictor (SRAMP, http://www.cuilab.cn/sramp/) database, with set the parameter to “Analyze RNA secondary structure” and the tissue parameter to “General (default)”.

### 2.14 Regulation network construction

The microRNAs (miRNAs) of biomarkers were predicted by miRWalk (http://mirwalk.umm.uni-heidelberg.de/) and TargetScan (http://www.targetscan.org/vert_72/) databases, and the intersection of the results predicted by 2 databases was taken as the key miRNA, and the key miRNA-mRNA network was visualized via Cytoscape software (v 3.10.2) (22). Transcription factors (TFs) for the hub genes were acquired from the Encyclopedia of DNA Elements (ENCODE, https://www.encodeproject.org/) database via NetworkAnalyst (http://www.networkanalyst.ca), and the hub genes-TF network was visualized via Cytoscape software (v 3.10.2).

### 2.15 Evaluation hub genes expression levels

A total of 15 tissue samples (5 control samples, 5 model samples, and 5 intervention samples) were acquired from the DCM model. Total RNA from the 15 samples were extracted with the TRIzol reagent (Ambion, USA) according to the manufacturer’s protocol. Then the RNA concentration was tested using NanoPhotometer N50. The cDNA was synthetized by reverse-transcribed using the SureScript-First-strand-cDNA-synthesis-kit, and the reverse-transcribed was performed with S1000™ Thermal Cycler (Bio-Rad, USA). The sequences of all primers can be found in **Supplementary Table S2**. The qPCR assay was performed with CFX Connect Real-time Quantitative Fluorescence PCR Instrument (Bio-Rad, USA) (pre-denaturation at 95℃ for 1 min, denaturation at 95℃ for 20s, annealing at 55℃ for 20s, extension at 72℃ for 30s, a total of 40 cycles). The relative quantification of mRNAs was calculated using the 2^-ΔΔCT^ method. The results from the RT-qPCR were exported to Excel, and then imported into Graphpad Prism 5 for statistical analysis and visualization.

### 2.16 Statistical analysis

Bioinformatics analyses were performed by R programming language (v 4.2.2). The statistical difference between two groups was assessed using a Wilcoxon test. The p < 0.05 was considered statistically significant.

## 3 Results

### 3.1 Effect of DAPA treatment on myocardial injury and fibrosis

**Figure 1:**
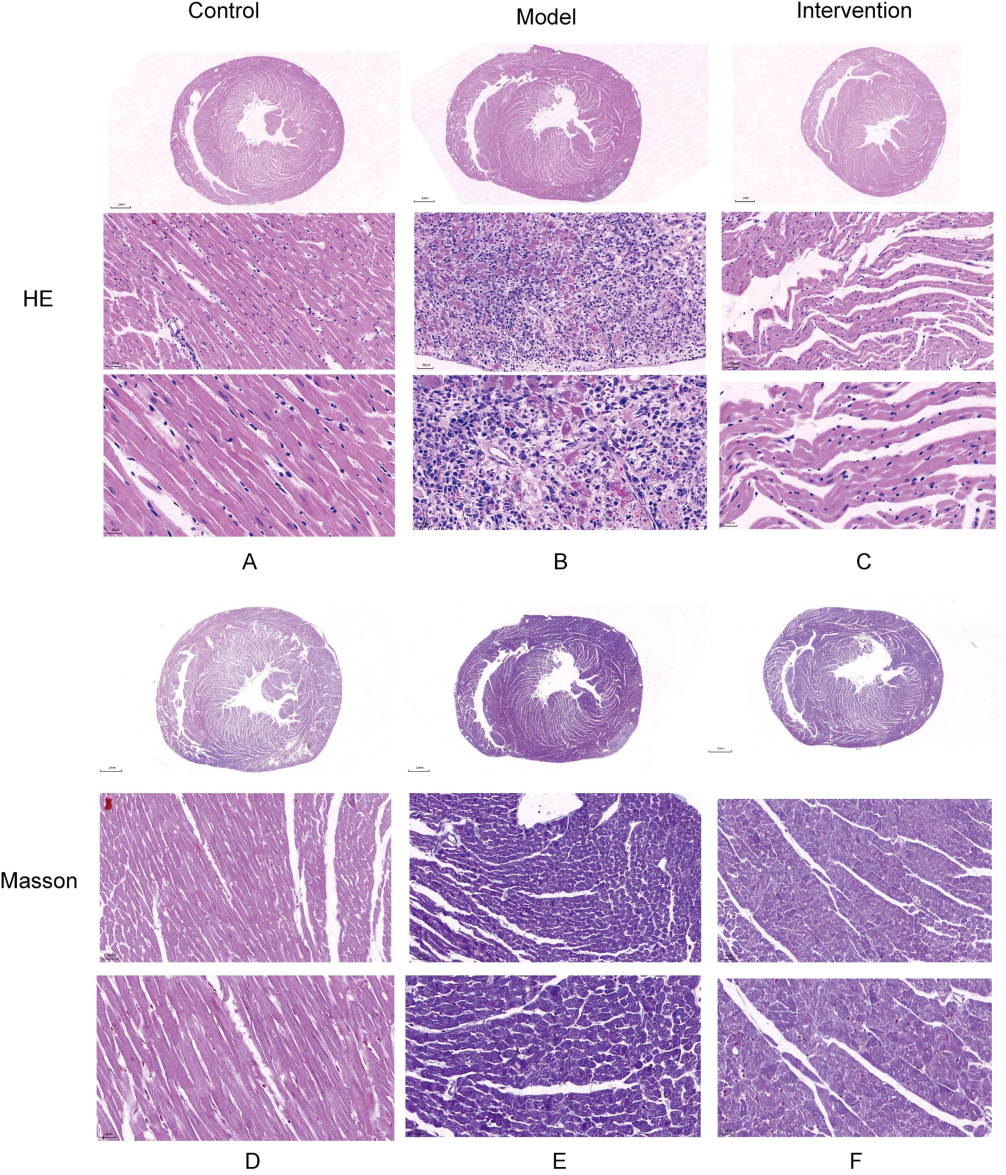
Histological analysis of myocardial injury and fibrosis in DCM rats. HE and Masson’s trichrome staining were used to assess the effects of DAPA on myocardial injury and fibrosis in DCM rats. **(A)** The control group shows normal cardiac structure, while **(B)** the model group exhibits disrupted muscle fibers and widened spaces. DAPA treatment **(C)** improves these changes with better-organized cardiac structure. Masson’s staining reveals normal collagen in controls **(D)**, increased fibrosis in the model group **(E)**, and reduced collagen deposition with DAPA treatment **(F)**. These results demonstrate that DAPA effectively alleviates myocardial injury and fibrosis in DCM rats.

HE staining results showed that in the control group, cardiac muscle fibers were well-organized, with no evidence of myocardial fiber damage and normal cellular spacing. In contrast, myocardial tissue in the model group exhibited significant damage, with some myofibrils dissolved, cardiac muscle fibers broken, and cellular spaces markedly widened. After DAPA treatment, rat cardiac muscle fibers appeared relatively well-aligned, with no fiber damage and normal cellular spacing, suggesting that DAPA played an important role in regulating myocardial injury and associated pathological processes (**Figures 1A–C**). Additionally, Masson staining revealed that in the model group, the degree of fibrosis in myocardial tissue was severe, with abundant collagen deposition. However, after DAPA treatment, the amount of collagen fibers in the myocardial tissue was significantly reduced, indicating that DAPA treatment effectively alleviated myocardial tissue fibrosis (**Figures 1D–F**).

### 3.2 Quality control analysis of sequencing data

**Figure 2:**
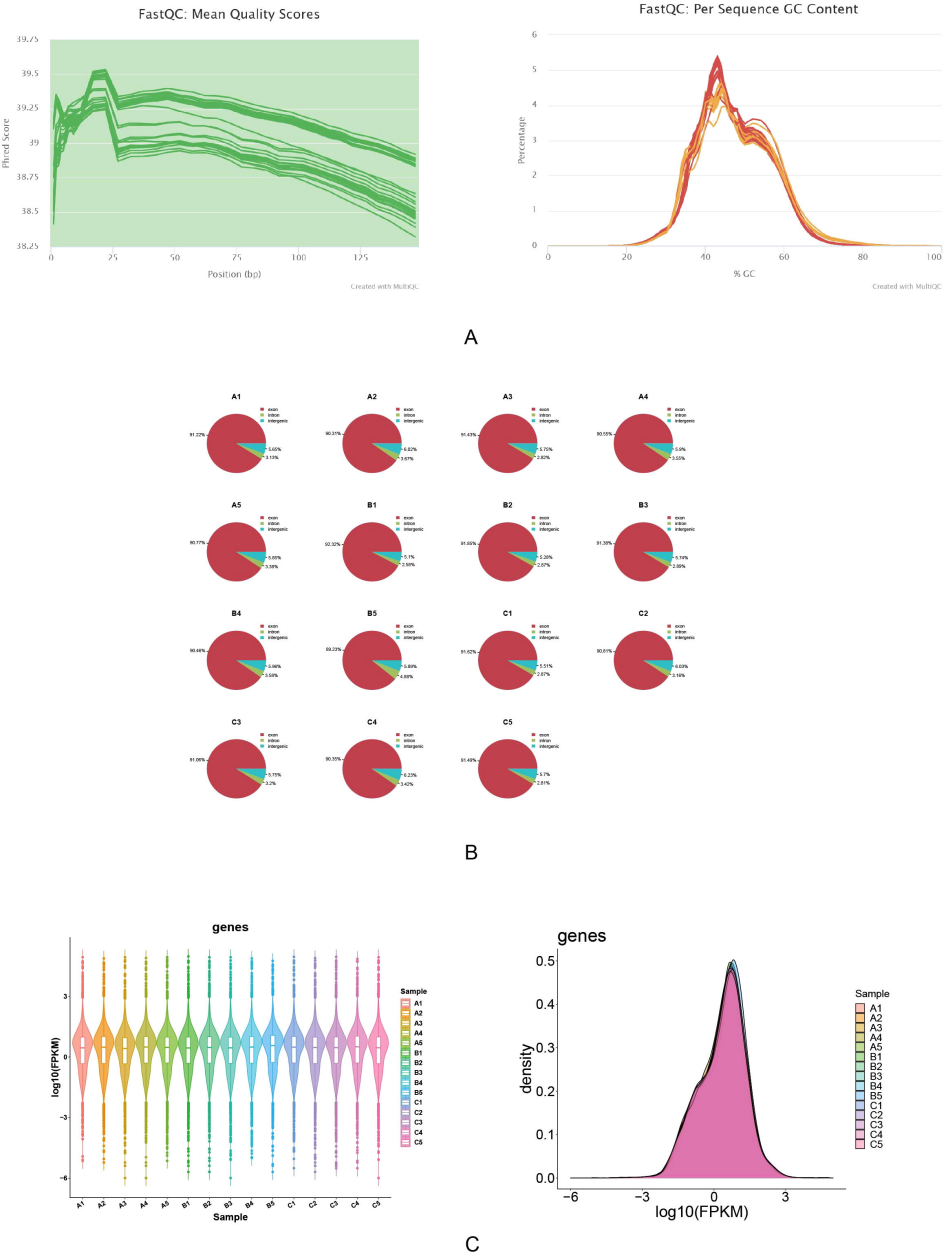
Quality assessment and gene expression analysis of RNA sequencing data. The quality of RNA sequencing data was evaluated using FastQC. **(A)** The mean quality scores across all bases remained high (>38), demonstrating excellent sequencing quality. **(B)** The per sequence GC content showed consistent distribution, with over 90% of sequences in each sample meeting quality standards. **(C)** Gene expression analysis indicated consistent distribution patterns across samples, with log10(FPKM) values primarily ranging from -3 to 3. These results confirm the high quality and reliability of the sequencing data.

The quality of the sequencing data was above Q30, and the GC content was uniformly distributed and concentrated in 40%-60%, with no GC offset, which indicated that the sequencing data had high accuracy and stable quality (**Figure 2A**). The probability of sequence matching to the exons region was above 89%, which indicated the high availability and reliability of the data, and laid the foundation for the subsequent gene expression analysis (**Figure 2B**). The range and pattern of distribution of log_10_ (FPKM) in different samples and the mean line on a straight line were shown by box plots, which indicated there was good comparability in gene expression among the different samples. And the log_10_(FPKM) density distribution plot was showed that log_10_(FPKM) formed a centrally distributed peak for all samples, which indicated that gene expression was not biased on the whole (**Figure 2C**).

### 3.3 Identification and functional enrichment analysis of 68 candidate genes

**Figure 3:**
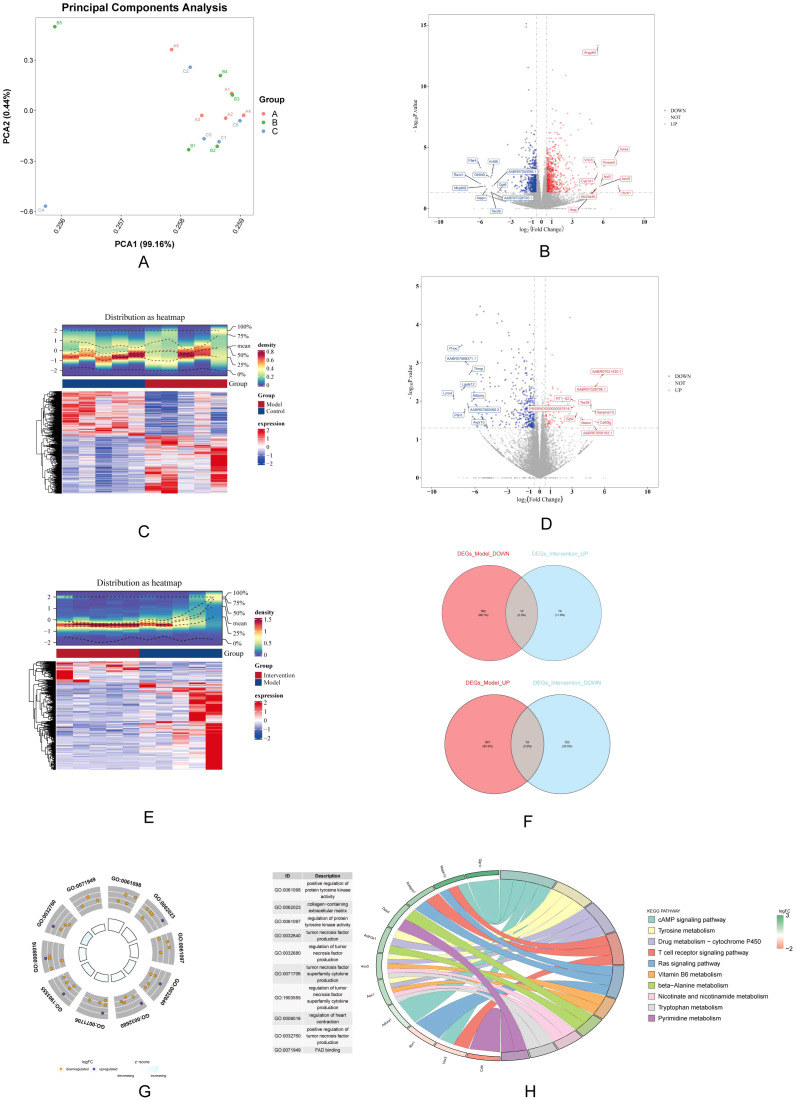
Differential gene expression analysis and functional enrichment. **(A)** PCA shows distinct clustering among the control, model, and DAPA groups, indicating clear separation of gene expression profiles. **(B)** Volcano plot displays significantly DEGs between model and control groups, highlighting upregulated (red) and downregulated (blue) genes. **(C)** Heatmap visualization illustrates the expression patterns of DEGs, with clear separation of groups. **(D)** A second volcano plot shows DEGs between the DAPA treatment and model groups. **(E)** The accompanying heatmap represents the distribution of DEGs, emphasizing the changes induced by DAPA. **(F)** Venn diagrams summarize the overlap of upregulated and downregulated genes across different comparisons. **(G)** GO analysis reveals significantly enriched biological processes. **(H)** KEGG enrichment analysis highlights pathways significantly associated with DEGs, detailing gene counts and statistical significance. These analyses underscore the molecular alterations and potential therapeutic targets in DCM treated with DAPA.

PCA result was showed significant differences between all 3 groups (**Figure 3A**). A total of 1,311 DEGs-Model were screened in model *vs.* control samples, comprising 734 genes (Angptl4, Tekt4, and Vnn3, etc.) with increased expression and 577 genes (Ffar4, Krt86, and Olr649, etc.) with decreased expression (**Figures 3B, C**). A total of 466 DEGs_Intervention were screened in intervention *vs.* model samples, comprising 91 genes (AABR07021430.1, AABR07028795.1, and RT1−S2, etc.) with increased expression and 375 genes (Pnoc, AABR07069371.1, and Thrsp, etc.) with decreased expression (**Figures 3D, E**). Subsequently, the 734 up-regulated genes in DEGs-Model with the 375 down-regulated genes in DEGs-Intervention and 577 down-regulated genes in DEGs-Model with the 91 up-regulated genes in DEGs-Intervention were intersected, resulting in the identification of 68 candidate genes (**Figure 3F**) (**Supplementary Table S3**). Furthermore, candidate genes were enriched and presented in 346 GO pathways, including 324 in biological process (BP), 12 in cellular component (CC), and 10 in molecular function (MF) (**Figure 3G**). For example, positive regulation of protein tyrosine kinase activity was enriched in BP; collagen-containing extracellular matrix was enriched in CC; FAD binding was enriched in MF. And the candidate genes were enriched and presented in 23 KEGG pathways (such as cAMP signaling pathway) (**Figure 3H** **).**

### 3.4 Identification and functional enrichment analysis of 2 hub genes

**Figure 4:**
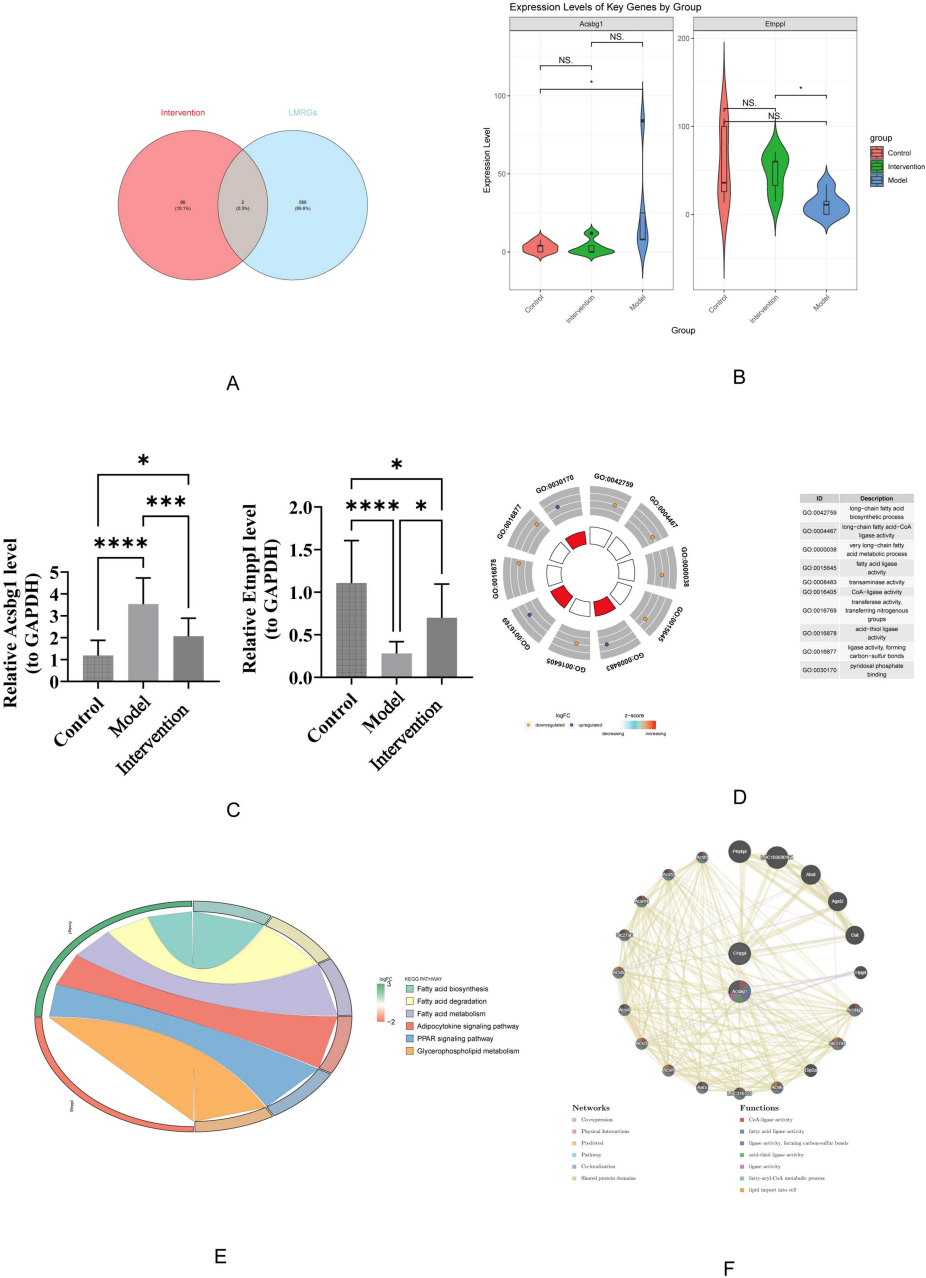
Key gene expression and functional enrichment analysis. **(A)** Venn diagram illustrating the overlap between intervention-related genes and LMRGs. **(B)** Violin plots show expression levels of key genes, Acsbg1 and Etnppl, across the control, model, and intervention groups, highlighting significant differences among the groups (p-values indicated). **(C)** Quantitative analysis confirms significant changes in gene expression levels between groups, with statistical significance denoted. **(D)** Gene Ontology (GO) enrichment analysis reveals that these key genes are primarily involved in fatty acid metabolism and regulation. **(E)** Further pathway analysis indicates significant enrichment in the fatty acid degradation, adipocytokine signaling, and PPAR signaling pathways. **(F)** Gene function network analysis illustrates complex interactions among these pathways. These results highlight the critical role of these key genes in mediating the effects of DAPA treatment in DCM.

The 68 candidate genes and 590 LMRGs were intersected, resulting in the identification of 2 hub genes (Acsbg1 and Etnppl) (**Figure 4A**). In which, compared with control group, Acsbg1 was significantly increase in model group (p < 0.05). In transcriptome sequencing data, the expression of Etnppl was significantly increase in intervention group compared with model group (p < 0.05) (**Figure 4B**). Likewise, RT-qPCR results showed Acsbg1 was obviously higher expression in model group compare to control groups and intervention group, while Etnppl was significantly lower expression in model group compare to control groups and intervention group (P < 0.05) (**Figure 4C**). Furthermore, the Acsbg1 and Etnppl were enriched and presented in 31 GO pathways, including 18 in BP (such as long-chain FA biosynthetic process) and 13 in MF (such as transaminase activity) (**Figure 4D**). And the Acsbg1 and Etnppl were enriched and presented in 6 KEGG pathways (such as FA biosynthesis) (**Figure 4E**). The GGI network results showed that the hub genes were functionally associated with 18 genes (Abat, Agxt2,and Oat, etc.) and predicted 7 functions (such as CoA-ligase activity) (**Figure 4F**).

### 3.5 GSEA of Acsbg1 and Etnppl

**Figure 5:**
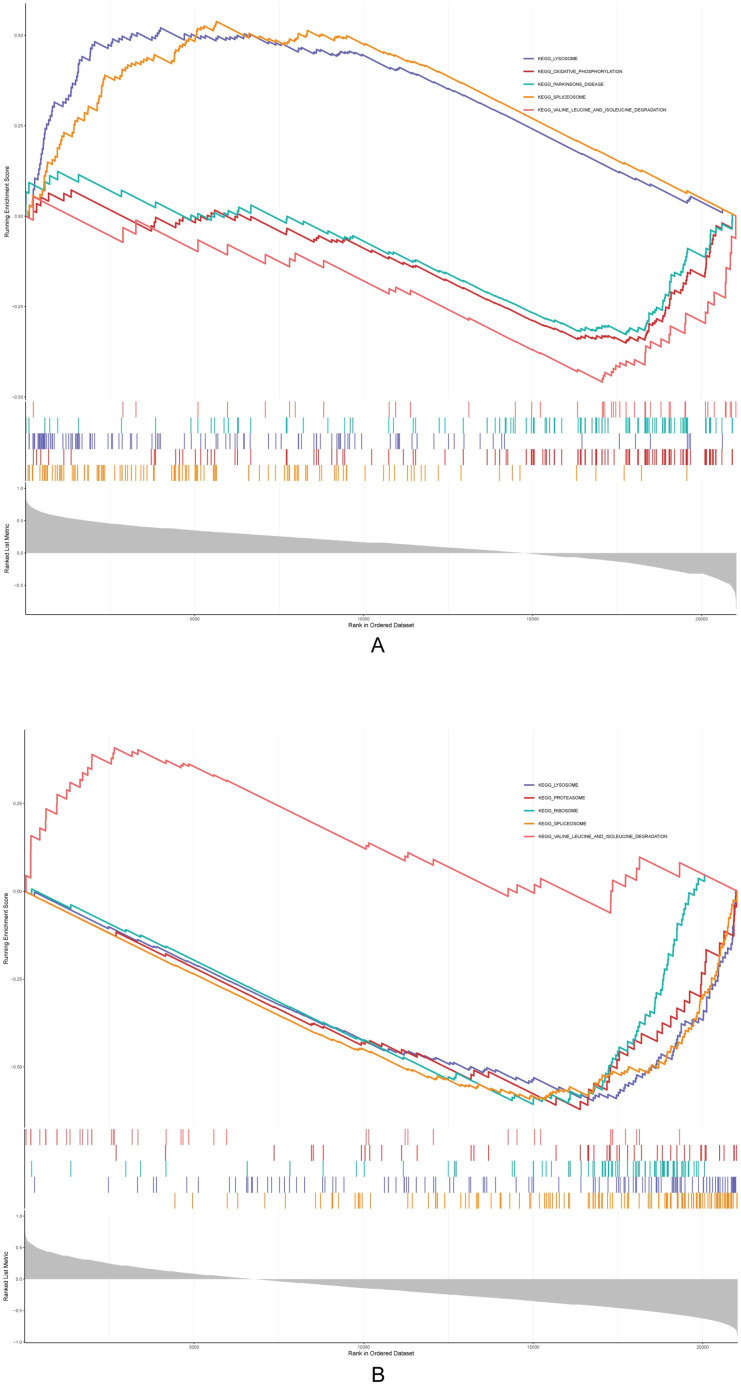
Gene set enrichment analysis (GSEA) of metabolic pathways. GSEA revealed significant enrichment of multiple metabolic pathways in the context of DAPA treatment. **(A)** Pathways related to lipid and glucose metabolism showed notable enhancement in the control group compared to the model group, particularly in fatty acid oxidation and glucose utilization processes. **(B)** Post-DAPA treatment, metabolic pathways demonstrated significant restoration, with marked enrichment in energy metabolism and fatty acid degradation pathways. These findings suggest that DAPA effectively modulates metabolic dysregulation in DCM, particularly impacting lipid and glucose metabolism.

The GSEA results showed that Acsbg1 was enriched to 13 KEGG pathways (lysosome, oxidative phosphorylation, and Parkinsons disease, etc.), and the Etnppl was enriched to 14 pathways (lysosome, proteasome, and ribosome, etc.) (**Figures 5A, B**). Among them, both Acsbg1 and Etnppl were significantly enriched for 14 pathways including spliceosome, lysosome, ribosome, proteasome, valine leucine and isoleucine degradation, N glycan biosynthesis, and DNA replication (**Supplementary Table S4**).

### 3.6 Predictive analysis of m6A binding sites for Acsbg1 and Etnppl

**Figure 6:**
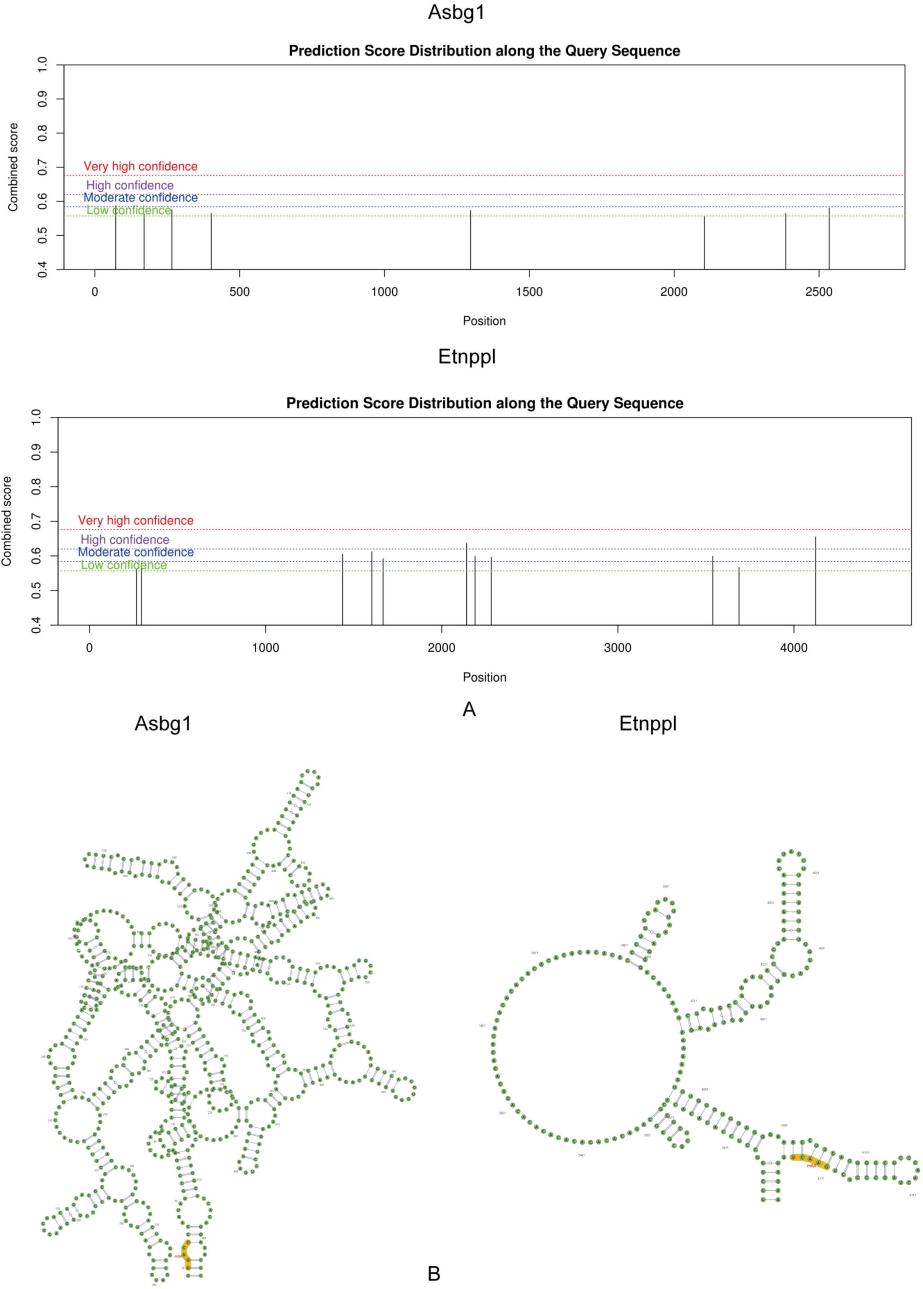
Prediction score distribution and secondary structure analysis of Acsbg1 and Etnppl. The prediction score distribution along the query sequences for Acsbg1 and Etnppl is depicted. **(A)** The score distribution for Acsbg1 reveals several high-confidence regions, indicating potential functional domains. **(B)** Etnppl also shows high-confidence predictions but with a distinct circular arrangement. The secondary structure analysis indicates that Acsbg1 exhibits a complex branching structure, while Etnppl is characterized by multiple protruding branches. Both proteins are noted for their conserved functional domains (highlighted in yellow), suggesting their important regulatory roles in metabolic processes.

The m6A binding sites for Acsbg1 and Etnppl were located at UCAGG in the RNA secondary structure. The m6A modification sites of Acsbg1 were predominantly located in regions of low confidence, while the m6A modification sites of Etnppl were mostly concentrated in regions of moderate confidence, with 2 located in high confidence regions (**Figures 6A, B**).

### 3.7 Regulation network of Acsbg1 and Etnppl

**Figure 7:**
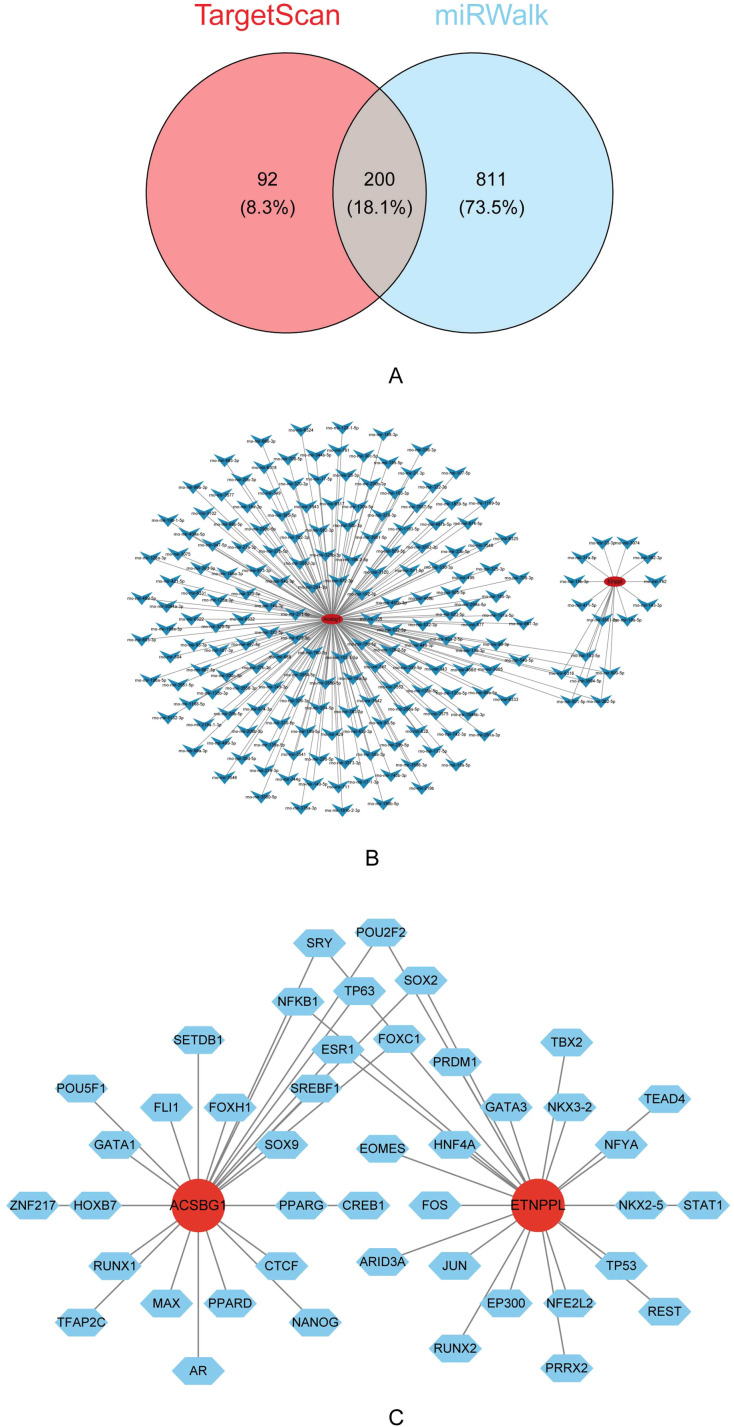
Regulatory mechanisms of key genes identified via bioinformatics analysis. **(A)** Venn diagram illustrating the overlap of predicted target genes between the TargetScan and miRWalk databases, revealing 200 shared genes (18.1%). **(B)** Interaction network analysis highlights extensive regulatory relationships among the predicted target genes. **(C)** Further transcription factor analysis identifies Acsbg1 and Etnppl as central regulatory nodes interacting with multiple transcription factors, including SOX2, GATA3, and PPARG. These findings suggest complex regulatory networks that underlie the therapeutic effects of DAPA in DCM.

A total of 292 miRNAs were predicted by TargetScan and 1,011 miRNAs were predicted by miRWalk were predicted by miRDB. The 200 key miRNA were taken from the intersection of the 2 database (**Figure 7A**). The key miRNA-mRNA regulatory network showed that the 184 key miRNAs were predicted by Acsbg1, and the 16 key miRNAs were predicted by Etnppl (**Figure 7B**). In which, rno-mir-153-5p, rno-mir-6316, rno-mir-500-5p, rno-mir-3594-5p, rno-mir-501-5p, and rno-mir-362-5p were co-predicted by Acsbg1 and Etnppl. Predicting the TFs of hub genes were important for probing the expression regulation mechanism of hub genes. In this study, 44 TFs were associated with Acsbg1 and Etnppl with potential regulatory relationships. Among them, FOXC1, ESR1, NF-κB1, TP63, SOX2, SRY, and POU2F2 were co-predicted by Acsbg1 and Etnppl. (**Figure 7C**).

## 4 Discussion

Lipid accumulation in the heart is an independent pathogenic event in the progression of DM, unrelated to other pathogenic factors such as ischemic, infectious, and rheumatic causes (23). Previous studies have shown that DAPA regulates blood lipids in diabetic patients to improve symptoms (24). Therefore, this study explores the therapeutic effects of DAPA on rats with DCM, identifying Acsbg1 and Etnppl as hub genes in the treatment process, and providing new reference points for subsequent clinical treatment research.

In this study, we established a DCM rat model and obtained myocardial tissue samples after DAPA intervention for sequencing, yielding stable and high-quality data. 1,311 genes were identified in the model group compared to the control group, and subsequently screened 68 candidate genes by intersecting DEG-model and DEG-control. Furthermore, we employed bioinformatics methods and intersected our findings with 590 LMRGs obtained from public databases, ultimately identifying Acsbg1 and Etnppl.

Etnppl is a gene that encodes the Etnppl protein, primarily expressed in the brain and liver, and is crucial for maintaining phospholipid homeostasis (25). In the Etnppl gene knockout mouse model constructed by Elmihi (26), increases in free glucose, total cholesterol, and very low-density lipoprotein (VLDL) were observed. This suggests that deletion of the Etnppl gene may lead to reduced expression of the LRP-1 gene in the liver, thereby decreasing VLDL uptake. Since the VLDL receptor is also expressed in heart tissue (27), which is consistent with our findings that in DCM rats treated with DAPA, increased expression of the Etnppl gene might improve myocardial lipid metabolism and alleviating the structural and functional disorders of DCM. Besides, research conducted by Wang et al. revealed that in a palmitic acid-induced diabetic mouse model, Etnppl suppresses autophagic flux through the activation of the ARG2/ROS signaling pathway, thereby mediating palmitic acid-induced hepatic insulin resistance (28). It is noteworthy that Xu et al. constructed a logistic regression model based on Etnppl to assess its clinical significance. Their findings indicated that the area under the AUC value was greater than 0.8, suggesting the model’s efficacy in differentiating between DCM and healthy control samples. This observation underscores the potential of Etnppl as a target for DCM research (29). Therefore, targeting Etnppl may offer a potential therapeutic strategy for DCM.

The Acsbg1 gene is located on chromosome 15 at q25.1 and encodes the Acsbg1 protein. Acsbg1 is a member of the long-chain acyl-CoA synthetase family (30). Studies have shown that Acsbg1 affects FA β-oxidation and disrupts energy metabolism, leading to cardiac metabolic disorders and increased risk of cardiovascular diseases (31). Furthermore, Acsbg1 may be involved in chronic inflammation, immune responses, and vascular abnormalities, which are crucial factors in disease progression (32). Acsbg1 is predominantly expressed in regulatory T (Treg) cells, particularly showing high levels in pulmonary ST2+ Treg cells. Its expression is regulated by TGF-β through the Smad2/3 signaling pathway. Through lipid metabolism regulation, Acsbg1 maintains immune tolerance and homeostasis, thereby suppressing inflammation. This anti-inflammatory function may potentially influence inflammation levels in cardiomyocytes affected by DCM (33).The present study constitutes the initial discovery of an association between Acsbg1 and DCM as a novel therapeutic target. Subsequent research will focus on the role of Acsbg1 in diseases such as DCM, with the objective of elucidating its underlying mechanisms. Furthermore, future studies will explore whether Acsbg1 exhibits a specific expression pattern in DCM and whether it correlates with the severity or progression of the disease.

Our study indicated a significant increase in the expression of the Etnppl protein in the intervention group, while the expression of Acsbg1 was markedly elevated in the model group. These up-regulated and down-regulated genes suggest that the expression of genes in the DCM heart undergoes substantial changes under the conditions of DAPA intervention. In summary, Acsbg1 and Etnppl are key regulators of lipid metabolism, which is closely linked to DCM. Therefore, targeting Acsbg1 and Etnppl could serve as a potential strategy for DAPA treatment of DCM.

The GO and KEGG enrichment analysis results of hub genes indicate their association with signaling pathways such as FA biosynthesis, FA degradation, and FA metabolism. One of the key characteristics of DCM is a disorder of energy metabolism, where toxic lipid metabolites accumulate in the diabetic heart a condition known as cardiac lipotoxicity (1). This may be related to increased uptake of FA by cardiomyocytes and/or reduced allosteric regulation of mitochondrial FA uptake, leading to incomplete FA oxidation. Excessive FA and FA-derived metabolites are the main pathogenic factors contributing to cardiac lipotoxicity in DCM (23, 34).

GSEA analysis revealed seven shared pathways between Acsbg1 and Etnppl, particularly the lysosomal pathway. Since excess saturated FA disrupt lysosomal function and increase protein toxicity in cardiomyocytes, we propose these hub genes may reduce lipid accumulation in DCM through enhanced lysosomal-mediated lipophagy, thereby improving FA metabolism (35, 36). Leucine accumulation, resulting from impaired BCAA metabolism, triggers both mTOR-mediated cell death and PPARα-enhanced FA oxidation, leading to increased cardiac lipid toxicity and vulnerability to ischemia-reperfusion injury (37). Another noteworthy pathway involves the proteasome, which plays a crucial role in cardiac function by clearing damaged proteins. In DCM, impaired proteasome activity leads to the accumulation of damaged proteins, resulting in proteotoxic stress and subsequent cardiac dysfunction (38). Related studies have shown that proteasome inhibition protects cardiac function by enhancing antioxidant gene expression and reducing oxidative damage and pathological remodeling (39). We hypothesized that Acsbg1 and Etnppl modulate these pathways in DCM pathogenesis. DAPA treatment appears to normalize both leucine metabolism and proteasome activity, potentially offering therapeutic benefits for DCM patients.

Subsequently, the hub genes-TFs regulatory networks for Acsbg1 and Etnppl were constructed to identify key TFs with potential regulatory roles. The cardioprotective effects of estrogen are well-established. The analysis of co-predicted TFs associated with hub genes, ESR1 emerged as a significant regulator of glucose homeostasis. ESR1 exerts its effects through dual regulation of solute carrier family 2 member 4 (Slc2a4), it enhances transcription through cooperative action with SP1 and CEBPA TF while inhibiting NF-κB activity, and promotes glucose transporter type 4 (GLUT4) translocation to the cell membrane. These mechanisms collectively contribute to improved insulin sensitivity (40). NF-κB is another crucial TF involved in DCM pathogenesis. It promotes disease development through two main mechanisms: inducing ferroptosis and mediating lipotoxicity-induced damage. These roles make NF-κB an important potential target for DCM therapeutic interventions (41). In summary, TFs such as ESR1 and NF-κB may influence DCM by regulating the transcription of Acsbg1 and Etnppl.

Studies have identified a strong involvement of pyroptosis in the progression of DCM (42), while m6A methylation acts as a crucial RNA epigenetic regulation mechanism (43). Meng et al. (44) discovered that METTL14 can suppress NLRP3-related pyroptosis in DCM by increasing the m6A methylation level of the TINCR gene, thereby reducing the mRNA stability of NLRP3. Previous research has also linked altered m6A modification patterns to myocardial fibrosis and myocyte hypertrophy in DCM (45). In the current study, the dysregulation of lipid and obesity-associated mRNAs in the DCM model might be related to altered m6A modifications of key genes (46). M6A sites were located at the UCAGG motifs in the RNA secondary structure, and suggest that DAPA might improve these differentially methylated sites directly or indirectly. However, further research is needed to confirm these findings. In this study, we employed bioinformatics approaches to analyze sequencing data and lipid metabolism-related genes, identifying two DCM-associated hub genes: Acsbg1 and Etnppl. Through comprehensive analyses including GSEA, m6A methylation profiling, and GGI network analysis, we further elucidated the molecular regulatory mechanisms of DAPA in DCM, providing a theoretical foundation for future investigations into the disease pathogenesis.

Despite the promising findings, our study has several limitations that warrant consideration. Due to limitations in terms of time, resources and other objective conditions, this study was unable to investigate the reversibility of the expression changes of Acsbg1 and Etnppl after the cessation of DAPA treatment. The significance of the reversibility of gene expression changes after DAPA treatment will be explored in future studies. To further validate and expand upon the current research results, it is planned that the relevant experiments will be repeated in a variety of different animal models in order to compare the intervention effect of DAPA on DCM in different animal models. At the same time, opportunities to collaborate with clinics will be actively sought in order to collect human tissue samples to investigate the mechanism and effect of DAPA on DCM from the human level. The small sample size of this study will be addressed by conducting large-scale sample analysis and introducing multi-sample validation experiments to enhance the extrapolation of results. Furthermore, the roles of other pathways in DCM will be explored to achieve a more comprehensive understanding of the pathogenesis of DCM. At the level of gene function research, the latest gene editing technology will be employed in combination with gene knockout experiments to provide a comprehensive analysis of the function of core genes in the pathogenesis of DCM. The development of DCM involves the abnormalities of many genes, and through gene knockdown, we can identify the specific roles of specific genes in the pathogenesis of DCM, which is of great significance.

## 5 Conclusion

In conclusion, this study successfully established a DAPA-treated DCM model and identified novel hub genes specifically associated with lipid metabolism in DCM pathogenesis. Through comprehensive functional prediction and systematic analysis of these genes, we have not only unveiled potential therapeutic targets for DAPA treatment but also provided valuable insights into the molecular mechanisms underlying DCM. These findings contribute significantly to our understanding of DCM pathophysiology and may pave the way for developing more effective therapeutic strategies for DCM patients.

## Data Availability

The datasets presented in this study can be found in online repositories. The names of the repository/repositories and accession number(s) can be found below: https://www.gsea-msigdb.org/gsea/msigdb/human/collections.jsp, MsigDB.
